# Cincinnati Again

**Published:** 1867

**Authors:** 


*Cincinnati Again.*—The *Lancet* is still melancholy, and can not be comforted at the lamentable condition of Rush College. We repeat, for its abundant solace, that the last session of Rush College lacked only two days of its usual number of working lecture days. In this institution holidays are scant and few—Christmas day and New Year's day only being allowed. The interim between these days is always occupied by lectures. We repeat, as many (if not more) lectures were given the last session in Rush College as were given in any four months course, in any college on the Continent. Believe us and rejoice, Dear *Lancet!* Rush College will go as far as the farthest in increasing the amount of time devoted to the lecture term—it will go as far as the farthest in insisting upon high attainments prior to graduation.

As an *Alma Mater* it looks with becoming pride upon its *alumni*, and challenges any other college to show a better record.

Preparatory schools like that at Ann Arbor, with expenses paid by State endowment, of course must be expected to consume a great deal of time in communicating professional *milk*—but students come to Chicago, or Cincinnati, so soon as they cut their molars, and are supposed capable of masticating professional *meat* in a reasonable period of time.

Six lectures a day are not too much for any healthy, adult intellect to receive and digest. The mind of the student is strengthened, his attention and perceptive faculties are disciplined, to an extent of which the puny dawdler over four lectures a day can have no conception.

The JOURNAL is in favor of lengthening the duration of the lecture term, but in no wise looks with complacency upon a proposition to diminish the number of daily lectures. Students come to Rush College to *work*, not to see city sights or luxuriate in holidays. We "indulge a trembling hope" it is so also in Cincinnati.

